# Salivary Flow Rate and Oral Status in Patients with Primary Sjögren’s Syndrome and Diffuse Cutaneous Systemic Sclerosis: A Cross-Sectional Study

**DOI:** 10.3390/diagnostics13061057

**Published:** 2023-03-10

**Authors:** Ana Glavina, Ivona Božić, Katica Parat, Dijana Perković, Dolores Biočina-Lukenda, Dušanka Martinović Kaliterna, Mislav Radić

**Affiliations:** 1 Dental Clinic Split, 21000 Split, Croatia; 2 Department of Oral Medicine and Periodontology, Study of Dental Medicine, School of Medicine, University of Split, 21000 Split, Croatia; 3 Division of Rheumatology and Clinical Immunology, Center of Excellence for Systemic Sclerosis in Croatia, University Hospital Split, 21000 Split, Croatia; 4 School of Medicine, University of Split, 21000 Split, Croatia

**Keywords:** Sjögren’s syndrome, diffuse cutaneous systemic sclerosis, dental caries, periodontal diseases, oral health

## Abstract

Determination of salivary flow rate and oral status in patients with primary Sjögren’s Syndrome (pSS) and diffuse cutaneous systemic sclerosis (dcSSc) and comparison with control subjects. Thirty-one pSS patients, 28 dcSSc patients, and 28 control subjects participated in this single-center, cross-sectional study. Unstimulated whole salivary flow rate (UWSFR) and stimulated whole salivary flow rate (SWSFR), salivary pH, DMFT index (D—decayed, M—missing, F—filled tooth), periodontal pocket depth (PPD), clinical attachment level (CAL), interincisal distance, and OHRQoL (oral health-related quality of life) were analyzed in all three groups of subjects. Primary SS and dcSSc patients had statistically significant lower values of UWSFR (0.20; 0.38 vs. 0.91 mL/min) and SWSFR (0.56; 0.70 vs. 1.64 mL/min) compared with control subjects (*p* < 0.001, Kruskal-Wallis test). Salivary pH values were statistically significantly lower in pSS and dcSSc patients compared with control subjects (6.00; 6.25 vs. 7.00, respectively) (*p* < 0.001, Kruskal-Wallis test). The DMFT index of dcSSc patients was higher (28.50) and statistically significant compared to control subjects (20.00) (*p* = 0.01). The prevalence of periodontitis was the same in pSS and dcSSc patients and control subjects (*p* = 0.384). Primary SS and dcSSc patients had a statistically significant decreased interincisal distance compared to control subjects (43.80; 38.00 vs. 48.00) (*p* = 0.003 and *p* < 0.001, respectively). Primary SS and dcSSc patients show decreased UWSFR and SWSFR, salivary pH values closer to an acidic medium, higher DMFT index, higher prevalence of periodontitis, decreased interincisal distance, and poorer OHRQoL, i.e., poor oral and periodontal health.

## 1. Introduction

Sjögren’s syndrome (SS) and systemic sclerosis (SSc) are systemic autoimmune diseases that can manifest in the oral cavity. Early recognition of symptoms and signs in the oral cavity is important, considering the morbidity and mortality of some systemic autoimmune diseases, such as SSc. Connective tissue diseases that manifest in the oral cavity include SS, discoid lupus erythematosus (DLE), systemic lupus erythematosus (SLE), SSc, dermatomyositis (DM), mixed connective tissue disease (MCTD), and rheumatoid arthritis (RA) [1]. The most important oral feature of primary Sjögren’s syndrome (pSS) is xerostomia. It results from an objectively decreased salivary flow. Hyposalivation is quantified by the sialometry test. Xerophthalmia is also present. Diagnosis of SS begins when the patient enters the outpatient/dental office. Inspection of patients with SS may reveal chronically enlarged major salivary glands with periods of acute exacerbation due to hyposalivation; chapped and dry lips; cheilitis angularis due to superinfection with C. albicans; and the lipstick sign, which indicates traces of lipstick on the labial surfaces of the incisors. Clinical oral examination reveals an extremely dry, erythematous mucosa that may be painful when touched with a dental mirror. The tongue is dry, erythematous, smooth, and wrinkled. Saliva is sticky and thick. Xerotrachea may also be present. Patients complain of difficulty speaking, chewing, swallowing, and wearing mobile prosthetic dentures. The most common clinical oral manifestations of pSS due to hyposalivation are glossitis, glossodynia, dysgeusia, oral candidiasis, cheilitis angularis, accelerated development of caries in physiologically clean areas (occlusal surfaces, neck of teeth), and acute infections of the major salivary glands [1].

SSc can be divided into limited cutaneous SSc (lcSSc) and diffuse cutaneous SSc (dcSSc) [2]. Inspection of patients with SSc may reveal: a face that looks similar to a mask; telangiectasia may appear on thin and stiff lips; and microstomia. Clinical oral examination often reveals telangiectasias on the hard palate mucosa. The tongue is hard and stiff. Patients complain of difficulty speaking and swallowing (motor dysfunction of the distal esophagus). Soft tissue involvement around the temporomandibular joint (TMJ) results in limited mobility of the mandible, i.e., pseudoankylosis. The masticatory muscles may be severely affected by fibrosis. This leads to resorption of the mandibular angle at the point where the masseter grips. Patients with SSc may develop gingival hyperplasia as a result of therapy with calcium channel blockers (BCCs). Pemphigus and lichenoid reactions may occur as a result of penicillamine therapy [1]. Hyposalivation is common in SSc patients due to concomitant secondary SS (sSS). As a result, hyposalivation leads to oral candidiasis, a higher incidence of caries (V. class) and periodontitis [1]. The localized form of scleroderma, i.e., morphea, manifests mainly on the skin and extremely rarely on the oral mucosa. En coup de sabre in linear scleroderma may manifest on the facial skin and result in facial hemiatrophy. A linear form of localized scleroderma may involve the periodontium of the tooth. Radiologically, a uniform widening of the periodontal ligament can be seen, especially around the posterior teeth, and it is present in less than 10% of patients. Soft tissue calcification is a characteristic radiological finding [1].

Numerous studies of systemic autoimmune diseases show worse oral health-related quality of life (OHRQoL) using various measurement instruments. A multicenter study by Fox et al. found that more than 96% of pSS patients had oral abnormalities. The most common subjective complaint was severe xerostomia [3]. In the study by Christensen et al. [4], the poorer oral health of pSS patients compared to control subjects was measured by the DMFT index (D—decayed, M—missing, F—filled tooth). The association between pSS and periodontitis remains controversial. Studies by Schiødt et al. [5] and Rhodus et al. [6] failed to confirm this association. Two studies by Baron et al. [7,8] showed hyposalivation, decreased interincisal distance, more missing teeth, and a higher prevalence of periodontitis in SSc patients. In these studies, decreased saliva production was associated with autoantibodies characteristic of SS (Ro 52/TRIM 21, SSA/Ro 60, SSB/La). Our study is the first to compare salivary flow rate, oral status, and OHRQoL in two systemic autoimmune diseases with oral manifestations (pSS and dcSSc). The unique aspect of our study is that we included dcSSc patients (compared to pSS patients) without concomitant sSS or autoantibodies associated with SS.

Thus, the objectives of our study are:

(1) to determine and compare the values of unstimulated whole salivary flow rate (UWSFR) and stimulated whole salivary flow rate (SWSFR) in patients with pSS, dcSSc, and control subjects,
(2) to determine and compare the dental and periodontal status, salivary pH, interincisal distance and OHRQoL in patients with pSS, dcSSc, and control subjects.

## 2. Materials and Methods

### 2.1. Study Design and Subjects

This was a single-center, cross-sectional study. The study was conducted from July 2018 to March 2020 in the Division of Rheumatology and Clinical Immunology, Center of Excellence for Systemic Sclerosis in Croatia, University Hospital Split, Split, Croatia, and in the Dental Clinic (teaching base of the School of Medicine, Study of Dental Medicine, University of Split, Split, Croatia). The study was performed after approval by the Ethics Committee of the Clinical Hospital Center Split, Split, Croatia (reference number 2181-198-03-04-16-0022). This study was conducted in accordance with the principles of the 1964 Declaration of Helsinki and its subsequent amendments. All subjects were enrolled after explanation of the study protocol and signing of the informed consent form. To ensure quality and transparency, the guidelines of the STROBE statement for cross-sectional studies were followed (Table S1) [9]. The study included 31 pSS patients, 28 dcSSc patients, and 28 control subjects. Six dcSSc patients were excluded from the study because they had concomitant sSS. All subjects were ≥18 years old.

The diagnosis of pSS was based on the 2016 American College of Rheumatology/European League Against Rheumatism (ACR/EULAR) classification criteria [10]. The final diagnosis of SSc was made according to the 2013 ACR/EULAR classification criteria [11]. The distinction between lcSSc and dcSSc was based on the criteria described by LeRoy et al. [2]. Diffuse cutaneous SSc was defined as proximal skin involvement (truncal and acral skin fibrosis) with an early and significant incidence of diffuse involvement [2]. The presence of concomitant sSS or autoantibodies associated with SS was the exclusion criterion in dcSSc patients. The diagnosis of sSS was based on the classification criteria of Shiboski et al. [10]. The co-author of this study (M. R.) performed a rheumatologic examination of pSS and SSc patients. An experienced periodontist, co-author of the study (K. P.), performed the initial periodontal examination, which included sialometric assessment, salivary pH, dental and periodontal status, interincisal distance, and OHRQoL.

The control group consisted of patients who had degenerative joint disease. Minor salivary gland biopsy (MSGB) was not performed in the control group, consistent with the guidelines of the local Ethics Committee. The pSS and dcSSc patients were matched for age and sex with the control subjects for comparison.

The exclusion criteria were:

- Medications (anticholinergics, tricyclic antidepressants, antihypertensives, antihistamines) causing salivary gland dysfunction,
- Systemic diseases (anorexia, bulimia, diabetes, HIV infection, hepatitis C) causing salivary gland dysfunction,
- Radiation therapy to the head and neck,
- Smoking.

### 2.2. Outcome Measures

Three sets of outcomes were obtained: 1. salivary flow rate (UWSFR, SWSFR), salivary pH, 2. oral and periodontal status, 3. interincisal distance, OHRQoL. UWSFR and SWSFR were collected in graduated tubes with a 1.5 cm diameter opening using the spit method. Sialometry was performed according to the guidelines of Navazesh [12]. Whole saliva was collected during the follicular phase of the cycle in fertile women. The pH of saliva was determined using indicator paper left in the oral cavity for one minute. We compared the color of the indicator paper with the color of the attached scale (Merck KGaA, Darmstadt, Germany). The pH reference value of saliva is 6.0 to 6.5. Saliva can be acidic or alkaline depending on whether the pH of saliva is below or above the reference value. Dental status was assessed using the DMFT index, excluding third molars [13]. If carious teeth were suspected, radiographic examination was performed in addition to the clinical examination. The initial periodontal examination was performed according to a standardized protocol using a dental mirror and a CPI (Community Periodontal Index) probe in daylight [14]. The applied probe force was equivalent to the weight of 25 g (0.025 kg × 9.81 m/s^2^). Data were recorded in the form recommended by the World Health Organization [15]. The CPI index values were as follows: 0—healthy periodontium, 1—gingival bleeding after probing, 2—calculus and bleeding, 3—shallow periodontal pockets (4–5 mm), and 4—deep periodontal pockets (6 mm and more). Periodontal pocket depth (PPD) was measured twice on all teeth. If the values obtained differed by >2 mm, the measurement was performed a third time, and the two closest values were retained. The PPD values at each site (mesio-buccal, mid-buccal, disto-buccal, mesio-lingual, mid-lingual, disto-lingual) were averaged. Clinical attachment level (CAL) was measured once for each tooth. The presence of periodontitis in a given tooth was defined as either PPD > 3 mm or CAL ≥ 5.5 mm [16,17]. The interincisal distance was measured at the beginning of the periodontal examination. This is important to avoid bias due to prolonged opening of the mouth. Patients were asked to open their mouths as wide as possible. The interincisal distance was measured as the distance from the incisal edge of the lower central incisor to the incisal edge of the upper central incisor [18]. Interincisal distance was measured in edentulous subjects with dentures in the mouth. The Croatian version of the Oral Health Impact Profile-49 (OHIP-49) was used to assess OHRQoL, without modifications relative to the published version [19].

### 2.3. Statistical Analysis

All analyzes were performed with IBM SPSS Statistics, version 22.0 (IBM Corp., Armonk, NY, USA). The distribution of continuous variables in the groups was expressed as mean ± standard deviation. When the distribution deviated from the normal, the median was used as the mean and the interquartile range (IQR) as the dispersion indicator. Normality was tested with the Kolmogorov-Smirnov test. The T-test was used for differences in numerically normally distributed values, whereas the Kruskal-Wallis test was used in the case of distribution deviation. Associations between qualitative variables were analyzed with the Chi-square (χ^2^) test, the χ^2^ test for a linear trend, or Fisher’s exact test, as appropriate. Linear correlations between continuous variables were evaluated with Spearman’s rank coefficient. *p*-values <0.05 were considered statistically significant in all data analyzes.

## 3. Results

### 3.1. Study Subjects

Three groups of subjects participated in the study: pSS patients (*n* = 31), dcSSc patients (*n* = 28), and control subjects (*n* = 28). There were no significant differences between the groups with regard to age and gender. Most pSS patients were women [96% women; mean age 53.12 (47.00–65.00)]. The mean age of dcSSc patients was 59.50 (49.00–69.00) years. Most of the patients were women (92.86%). The mean age of the control subjects was 53.50 (48.00–61.50) years, and most of them were also women. A comparison of the observed variables between pSS and dcSSc patients and control subjects is shown in Table 1.

### 3.2. Sialometric Evaluation

**Figure 1:**
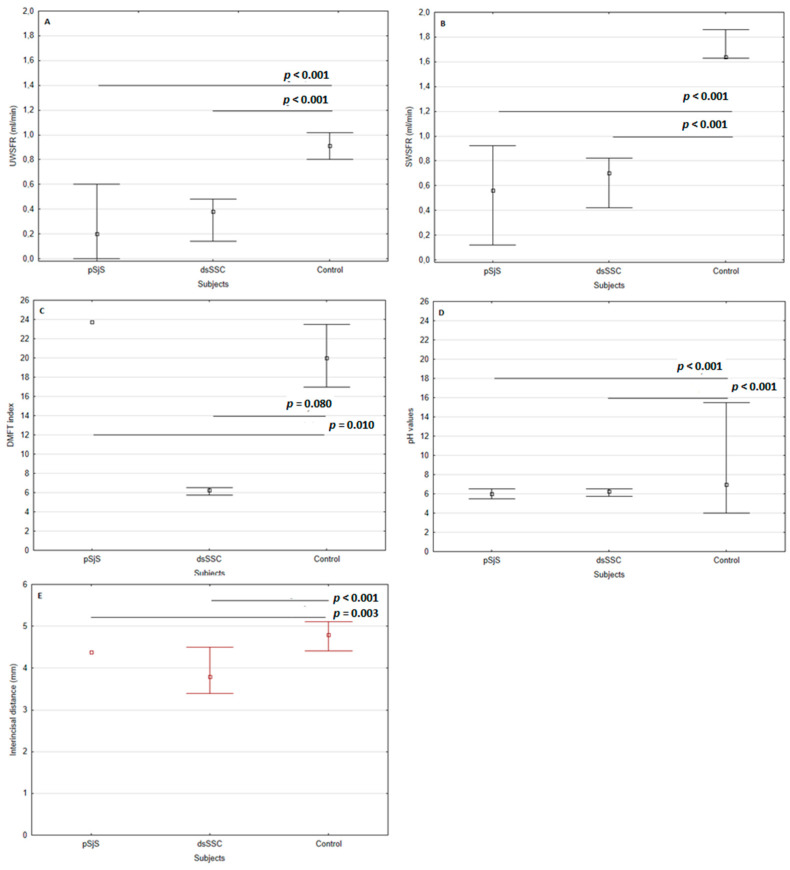
Comparison of variables in pSS and dcSSc patients: (**A**) UWSFR, (**B**) SWSFR, (**C**) DMFT index, (**D**) pH values of saliva, (**E**) interincisal distance (mm). Abbreviations: pSS, primary Sjögren’s Syndrome; dcSSc, diffuse cutaneous systemic sclerosis; UWSFR, unstimulated whole salivary flow rate; SWSFR, stimulated whole salivary flow rate; DMFT, D—decayed, M—missing, F—filled tooth.

Primary SS and dcSSc patients had statistically significant lower values of UWSFR (0.20; 0.38 mL/min) and SWSFR (0.56; 0.70 mL/min) compared with control subjects (UWSFR 0.91 mL/min; SWSFR 1.64 mL/min) (*p* < 0.001, Kruskal-Wallis test) (Figure 1A,B). Age was not a confounder for sialometric assessment between groups.

### 3.3. Oral and Periodontal Status

Mean salivary pH was statistically significantly lower in pSS and dcSSc patients compared with control subjects (6.00 and 6.25 vs. 7.00, respectively) (*p* < 0.001) (Figure 1D). The mean DMFT index of the pSS patients was 23.74 ± 7.28 compared with the control subjects (20.00), and this difference was not statistically significant (*p* = 0.08). The DMFT index of the dcSSc patients was statistically significantly higher (28.50) compared with the control subjects (20.00) (*p* = 0.01) (Figure 1C). The prevalence of periodontitis was the same in pSS and dcSSc patients (83.87%; 85.71%) as in control subjects, and this difference was not statistically significant (*p* = 0.348). Primary SS and dcSSC patients had a statistically significant decreased interincisal distance compared to control subjects (43.80 and 38.00 vs. 48.00, respectively) (*p* = 0.003 and *p* < 0.001) (Figure 1E).

### 3.4. OHRQoL

**Figure 2:**
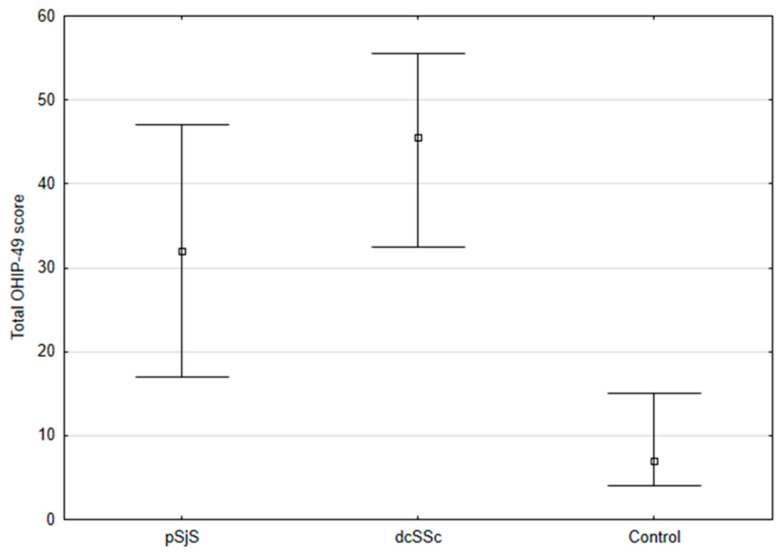
Total OHIP-49 score in pSS and dcSSc patients (*p* < 0.001). Abbreviations: pSS, primary Sjögren’s Syndrome; dcSSc, diffuse cutaneous systemic sclerosis; OHIP-49, Oral Health Impact Profile-49.

The mean OHIP-49 total score was statistically significantly higher in pSS and dcSSc patients compared with control subjects (32.00 and 45.50 vs. 7.00, respectively) (*p* < 0.001, Kruskal-Wallis test) (Figure 2). A comparison of the OHIP-49 subscale items between pSS and dcSSc patients and control subjects is shown in Table 2.

## 4. Discussion

Connective tissue diseases manifesting in the oral cavity include SS, discoid lupus erythematosus (DLE), systemic lupus erythematosus (SLE), SSc, dermatomyositis (DM), mixed connective tissue disease (MCTD), and rheumatoid arthritis (RA) [1]. Hyposalivation is one of the most common oral manifestations in patients with pSS and dcSSc. In pSS patients, it is part of the etiopathogenetic mechanism of the disease, i.e., the lacrimal and salivary glands are most commonly affected by the disease. In dcSSc patients, it is attributed to concomitant sSS. There are few studies that have investigated salivary function in dcSSc patients without concomitant sSS or SS-associated autoantibodies. The study by Parat K et al. talks about the relationship between hyposalivation and disease activity and severity in these patients [20]. The authors Baron et al. [7] and Chu et al. [21] found statistically significant lower sialometry values in SSc patients (*p* = 0.0259 and *p* < 0.01, respectively). These results are consistent with the results of our study. The advantage of our study was that we included dcSSc patients without concomitant sSS or SS-associated autoantibodies. The results of our study demonstrate the importance of oral health screening in SSc patients. Further studies should focus on finding the most relevant method to assess the oral component (xerostomia) of SSc patients, as well as ultrasound examinations of the major salivary glands. This is important for the possible inclusion of the oral cavity in the classification criteria. Compared to dcSSc patients, pSS patients had lower UWSFR and SWSFR. This can be explained by the etiopathogenetic mechanism of SS, i.e., focal lymphocytic infiltration of the salivary glands. The UWSFR in pSS patients in our study is consistent with the systematic review of the literature and meta-analysis by Martínez-Ceballos et al. (0.18 mL/min) [22].

Salivary pH values were mostly within the reference range in the pSS and dcSSc patients. However, it should be noted that salivary pH values were closer to the acidic medium in both groups. Salivary pH values are lower in pSS patients compared to dcSSc patients. This may be explained by lower sialometry values in pSS patients compared to dcSSc patients, i.e., lower buffering capacity of saliva.

The systematic review by Maarse et al. [23] confirms with statistical significance higher DMFT and DMFS indexes in pSS patients compared to control subjects. Authors Christensen et al. [4] (*p* < 0.05 and *p* < 0.001, respectively), Pedersen et al. [24] (*p* < 0.001), and Márton et al. [25] (*p* < 0.05) obtained similar results in pSS patients. However, the pSS patients in our study had a higher DMFT index, although without statistical significance. These results are likely due to a smaller sample size. The study by Wood et al. [26] showed a statistically significant higher DMFS index in 31 SSc patients. This was confirmed by the results of our study. Compared to pSS patients, dcSSc patients have a higher DMFT index, i.e., worse dental status. Given the lower values of sialometry and salivary pH in pSS patients compared to dcSSc patients, we would not expect such results. However, there are several possible explanations for the better oral status of pSS patients. The oral component is part of the classification criteria for the diagnosis of SS. Therefore, pSS patients are more likely to see an oral medicine specialist because of the need for collaboration between rheumatologists and oral medicine specialists in making the final diagnosis. Thus, pSS patients are educated about protecting their teeth and preventing caries, as well as protecting soft oral tissues and methods to reduce dry mouth. Better collaboration between rheumatologists and oral medicine specialists leads to better oral status in pSS patients, i.e., better OHRQoL. The oral component in SSc patients is neglected. This is evident from the higher DMFT index in our study compared to pSS patients and control subjects. The reasons for this could be the following: oral component is not part of the classification criteria for the diagnosis of SSc, oral health is neglected due to severe systemic complications, irregular dental follow-up, decreased interincisal distance, and limited motor mobility of the arms.

The question of whether SS and SSc patients are at higher risk for periodontitis remains controversial. The systematic review by Maarse et al. [23] showed that SS patients are not at higher risk for developing periodontitis. The meta-analysis by Wu et al. [27] showed that there was no difference between PPD and CAL in SS patients and control subjects. Studies by Baron et al. [7], Wood et al. [26] and Leung et al. [28] showed a statistically significant higher prevalence of periodontitis in SSc patients (*p* < 0.0001, *p* < 0.001, *p* < 0.001). Studies by Chu et al. [21] and Nagy et al. [29] have shown similar prevalence, but without statistical significance. Their results are consistent with the results of our study. The heterogeneity of the results can be explained by different classification criteria of periodontitis, so the studies cannot be compared. Most studies, such as ours, have a small sample size. The etiology of periodontitis is multifactorial. The results obtained show that periodontitis should not go unrecognized and untreated in pSS and SSc patients. The negative effects of periodontitis on OHRQoL are well known. Periodontitis is associated with a number of systemic diseases and conditions: adverse pregnancy outcomes, cardiovascular disease (CV), type II diabetes mellitus (DM), respiratory disease, fatal pneumonia in hemodialysis patients, chronic kidney disease, metabolic syndrome, RA. The bidirectional relationship between periodontitis and DM is well known, i.e., treatment of periodontitis improves glycemic control in DM patients [30]. The relationship between periodontitis and RA is particularly interesting, and many studies address its bidirectional role. Although they are etiopathogenetically completely different diseases, there is evidence that both periodontitis and RA are characterized by persistent levels of proinflammatory cytokines and associated molecules. The idea is that polymorphisms of genes encoding specific cytokines that lead to connective tissue damage and alterations in bone metabolism in the two aforementioned pathologies provide a bridge between these diseases [31]. Therefore, the high prevalence of periodontitis in our study demonstrates the importance of regular dental follow-up in pSS and dcSSc patients. A periodontist should also be part of the multidisciplinary medical team caring for SS and SSc patients. Regular dental follow-ups are required at least four times a year for pSS and dcSSc patients to professionally remove plaque and to detect caries early. Depending on the dentist’s assessment and taking into account sialometric values, they may be performed more frequently. Oral health education, improvement, and adaptation of oral hygiene to the specific needs of SS and dcSSc patients prevent the progression of periodontitis, leading to tooth loss and decreased OHRQoL. This includes the use of proper toothbrushing techniques, daily use of interdental brushes, topical fluoride therapy in the form of gel, and the use of oscillating-rotating-pulsating toothbrushes in dcSSc patients due to hand involvement.

Authors Wood et al. [26], Nagy et al. [29], Chu et al. [21], Baron et al. [7], and Hudson et al. [32] showed statistically significant decreased interincisal distance in SSc patients compared to control subjects. These results are consistent with the results of our study, which showed an average interincisal distance of 38.00 mm in dcSSc patients. In our study, dcSSc patients had a smaller interincisal distance compared with pSS patients. This result is expected considering the etiopathogenetic mechanism of SSc, i.e., perioral soft tissue fibrosis. The decreased interincisal distance in pSS patients compared with control subjects is probably due to the discomfort caused by the presence of dry mouth. This is confirmed by the lower UWSFR and SWSFR in pSS patients compared to dcSSc patients and control subjects.

The results of our study showed that pSS and dcSSc had a negative effect on OHRQoL. Moreover, the OHIP-49 total score of dcSSc patients was 14 points higher than that of pSS patients, indicating worse OHRQoL in dcSSc (*p* < 0.001). These results are consistent with those of Baron et al. [7], who found a 15 points higher total OHIP score (*p* < 0.0001) in SSc patients compared with control subjects. Therefore, assessment of the psychological aspects of these two systemic autoimmune diseases must be part of the comorbidities in a multidisciplinary approach.

The presence of SS in patients with SSc appears to have a greater association with lcSSc phenotype [33,34]. The advantage of our study is that the bias was reduced by including dcSSc patients (as a specific subtype of autoimmunity) who did not have concomitant sSS or autoantibodies associated with SS. This suggests that hyposalivation in dcSSc patients is due to glandular fibrosis. The disadvantages of our study are the small sample size (a relative limitation, as dcSSc is a rare disease) and, consequently, the lack of multivariate regression analysis. We used the Croatian version of the OHIP-49 to assess OHRQoL, which has not been validated for pSS and SSc patients. Further comprehensive studies of oral status in systemic autoimmune diseases are needed and should be performed with comparable measurement instruments and exclusion criteria.

The results of our study show the importance of involving different dental specialists (oral medicine specialists, periodontists) in the treatment protocols for systemic autoimmune diseases such as pSS and SSc. This is important because the association between oral and systemic health is well known. This approach leads to better OHRQoL, but also better overall survival for patients with systemic autoimmune diseases.

## Figures and Tables

**Table 1:** Characteristics of pSS and dcSSc patients and control subjects.

Variable	pSS Patients (*n* = 31)	dcSSc Patients (*n* = 28)	Control Subjects (*n* = 28)	*p*-Value
**Age (years)**	53.12 (47.00–65.00)	59.50 (49.00–69.00)	53.50 (48.00–61.50)	>0.05
**UWSFR (mL/min)**	0.20 (0.00–0.60)	0.38 (0.14–0.48)	0.91 (0.80–1.02)	<0.001
**SWSFR (mL/min)**	0.56 (0.12–0.92)	0.70 (0.42–0.82)	1.64 (1.43–1.86)	<0.001
**pH values**	6.00 (5.50–6.50)	6.25 (5.75–6.50)	7.00 (4.00–15.50)	<0.001
**DMFT index**	23.74 (20.00–26.50)	28.50 (24.00–32.00)	20.00 (17.00–23.50)	0.08, 0.01
**Interincisal distance (mm)**	43.80 (40.00–49.00)	38.00 (34.00–45.00)	48.00 (44.00–51.00)	0.003, <0.001

Data expressed as median and interquartile range (IQR). Age is not a confounder for sialometric assessment between groups. Abbreviations: pSS, primary Sjögren’s Syndrome; dcSSc, diffuse cutaneous systemic sclerosis; UWSFR, unstimulated whole salivary flow rate; SWSFR, stimulated whole salivary flow rate; DMFT, D—decayed, M—missing, F—filled tooth.

**Table 2:** OHIP-49 scores in pSS and dcSSc patients, and control subjects.

	pSS Patients (*n* = 31)		dcSSc Patients (*n* = 28)		Control Subjects (*n* = 28)		*p*-Value
	Median	IQR	Median	IQR	Median	IQR	
**Total OHIP-49 score**	32.00	(17.00–47.00)	45.50	(32.50–55.50)	7.00	(4.00–15.00)	<0.001
**OHIP subscales**							
1. Functional limitation	8.00	(5.00–12.00)	15.00	(12.00–17.00)	1.00	(0.00–6.00)	<0.01
2. Physical pain	6.00	(4.00–7.00)	9.00	(6.00–12.00)	2.00	(0.00–5.00)	0.001
3. Psychological discomfort	6.00	(4.00–8.00)	8.00	(4.00–9.00)	5.00	(3.00–8.00)	0.353
4. Physical disability	4.00	(2.00–9.00)	13.00	(9.00–19.00)	0.00	(0.00–2.00)	<0.01
5. Psychological disability	1.00	(0.00–5.00)	6.00	(5.00–8.00)	0.00	(0.00–0.00)	0.007
6. Social disability	0.00	(0.00–2.00)	3.00	(0.00–4.00)	0.00	(0.00–0.00)	0.003
7. Handicap	0.00	(0.00–4.00)	4.00	(0.00–6.00)	0.00	(0.00–0.00)	<0.01

Kruskal-Wallis test. Data expressed as median and interquartile range (IQR). Abbreviations: pSS, primary Sjögren’s Syndrome; dcSSc, diffuse cutaneous systemic sclerosis; OHIP-49, Oral Health Impact Profile-49.

## Data Availability

Data are available at the corresponding author’s e-mail upon request.

## Supplementary Materials

The following supporting information can be downloaded at: https://www.mdpi.com/article/10.3390/diagnostics13061057/s1, Table S1: STROBE Statement—Checklist of items that should be included in reports of cross-sectional studies.
